# Sexual risk behavior among people living with HIV and AIDS on antiretroviral therapy at the regional hospital of Sokodé, Togo

**DOI:** 10.1186/1471-2458-14-636

**Published:** 2014-06-22

**Authors:** Issifou Yaya, Bayaki Saka, Dadja Essoya Landoh, P'Niwè Massoubayo Patchali, Makawa-Sy Makawa, Sékandé Senanou, Daoudou Idrissou, Bassan Lamboni, Palokinam Pitche

**Affiliations:** 1 Laboratoire de Santé Publique (EA 3279), Aix-Marseille Université, Marseille, France; 2 Service de dermatologie et IST, CHU Sylvanus Olympio, Université de Lomé, Lomé, Togo; 3 Division de l’épidémiologie, Ministère de la santé, BP: 1396 Lomé, Togo; 4 Centre Hospitalier Régional (CHR) de Sokodé, Service de dispensation d’antirétroviraux (ARV), Sokodé, Togo; 5 Ministère de la Santé, Direction Préfectorale de la Santé de Tône, Dapaong, Togo; 6 Centre Hospitalier Régional (CHR) de Kara-Tomdè, Service de Médecine générale, Kara, Togo; 7 Programme National de lutte contre les maladies non transmissibles, Ministère de la Santé, Lomé, Togo; 8 Conseil National de Lutte contre les IST/VIH/Sida, Lomé, Togo

**Keywords:** Sexual risk behavior, Antiretroviral therapy, People living with HIV and AIDS, Togo

## Abstract

**Background:**

Several studies on the sexual risk behaviors in sub-Saharan Africa have reported that the initiation of antiretroviral therapy leads to safer sexual behaviors. There is however a persistence of risky sexual behavior which is evidenced by a high prevalence of sexually transmitted infections among people living with HIV and AIDS (PLWHA). We sought to determine the factors associated with risky sex among PLWHA on antiretroviral therapy in Togo.

**Methods:**

An analytical cross-sectional survey was conducted from May to July 2013 at regional hospital of Sokodé, Togo, and targeted 291 PLWHA on antiretroviral therapy for at least three months.

**Results:**

From May to July 2013, 291 PLWHA on antiretroviral treatment were surveyed. The mean age of PLWHA was 37.3 years and the sex ratio (male/female) was 0.4. Overall, 217 (74.6%) PLWHA were sexually active since initiation of antiretroviral treatment, of which, 74 (34.6%) had risky sexual relations. In multivariate analysis, the factors associated with risky sex were: the duration of antiretroviral treatment (1 to 3 years: aOR = 27.08; p = 0.003; more than 3 years: aOR = 10.87; p = 0.028), adherence of antiretroviral therapy (aOR = 2.56; p = 0.014), alcohol consumption before sex (aOR = 3.59; p = 0.013) and level of education (primary school: aOR = 0.34 p = 0.011; secondary school: aOR = 0.23 p = 0.003; high school: aOR = 0.10; p = 0.006).

**Conclusion:**

There was a high prevalence of unsafe sex among PLWHA receiving ART at the hospital of Sokodé. Factors associated with sexual risk behaviors were: low education level, non-adherence to ART, alcohol consumption before sex and the duration of ART. It is important to strengthen the implementation of secondary prevention strategies among this population group.

## Background

At the end of 2012, an estimated 35.3 million persons were living with HIV and AIDS (PLWHA) globally; 71% lived in sub-Saharan Africa where three quarters of all deaths due to AIDS in 2012 had occurred [1]. In Togo, the prevalence of HIV infection in the general population was estimated at 3.2% in the same year [2]. Although this prevalence is relatively low in comparison to other sub-Saharan Africa countries, it masks many disparities that may exist between population groups, regions and by gender. Antiretroviral therapy (ART) with sustainable prevention interventions (such as condom provision and health promotion) is associated with a decline in the HIV-related morbidity and mortality among PLWHA, improved life expectancy and quality of life [3]. As such, new issues have emerged including those concerning patients’ adherence to ART and their sexual behaviors [4].

Sexual risk behavior among people receiving ART is a major public health concern not only because of risks of HIV transmission, but also the potential risk of transmission of resistant strains [4].

In sub-Saharan Africa, several studies on sexual risk behavior were conducted in cohorts of PLWHA, who often were on ART. Most studies were carried out in East and South Africa [5-8] and a few in West Africa [9,10]. These studies have shown that PLWHA are highly sexually active. Some studies have compared the sexual behavior of PLWHA before and after initiation of ART [7,9] or compared those receiving ART to those not receiving treatment [5,6]. A study conducted in Côte d’Ivoire reported that the initiation of ART was associated with risky sexual behavior [9]. In Uganda a study [8] reported that in a population of PLWHA on ART, alcohol consumption and ignorance of HIV sero-status of the sexual partner predisposed people to risky sexual behaviors.

Other studies [6,11] reported ART initiation actually promoted safe sexual behaviors among PLWHA. Nevertheless, persistence of risky sexual behavior among PLWHA is evidenced by a high prevalence of sexually transmitted infections (STIs) among these populations [9,12].

Complementary studies are currently absent among PLWHA on ART and living in Togo. In order to implement relevant and specific interventions to prevent secondary sexual transmission of HIV in Togo, we conducted this study to document the factors associated with sexual risky behaviors among PLWHA on ART.

## Methods

### Study design

We conducted an analytical cross-sectional survey at regional hospital of Sodoké, Togo over a period of May to July 2013.

### Setting

The regional hospital of Sokodé is a health reference center of the central region which is one of the six health regions of Togo. The hospital of Sokodé is located about 350 km from the capital city Lomé. It serves four health districts with a total population of 654,074 inhabitants in 2013 [13]. In Togo, the central health region countered for 1,869 PLWHA and the hospital of Sokodé is the main site where 45% of these PLWHA are treated and followed up [14].

### Study population and sampling

We used a purposive and comprehensive sampling to include PLWHA aged 15 years or older, who were taking ART for at least three months and were followed up by the regional hospital of Sokodé. Of 843 PLWHA who were followed up in the regional hospital, 798 were aged over 15 years of age [14]. Of 458 PLWHA on ART who were seen during the study period in the hospital, 317 consented to participate to the study and, of these, 291 PLWHA completed the interview.

### Data collection

Data were collected through a questionnaire-assisted interview in French or in the local language, conducted by the clinic officers to ensure good understanding of the questions.

The questionnaire included socio-demographic information, clinical features, information on the adherence to ART, information on HIV/AIDS knowledge and aspects of sexual behavior. Sexual activity was defined as reporting at least one sexual partner during the previous 3 months.

To measure adherence to treatment, two methods were used, including: timely attendance at appointments for delivery of antiretroviral drugs and counting remaining tablets at the appointment for renewal of the order.

A patient was considered to have good adherence to antiretroviral treatment if he had a percentage of drug intake greater than or equal to 95%. We defined risky sex as unprotected sex with a partner of negative or unknown HIV status within three (3) months preceding the survey.

### Data analysis

Data entry was performed using Epidata software version 3.1 and analyzed in SPSS Inc version 17.0 (SPSS Inc, Chicago, IL, USA).

For continuous variables, mean and standard deviation were calculated while for categorical variables we calculated proportions. Our primary outcome of interest was having risky (unsafe) sex. Pearson chi-square test or Fisher′s exact test were used when appropriate in bivariate analysis.

Multivariate backwards stepwise logistic regression analysis was performed to identify independent risk factors for the primary outcome of having or not having risky sex. All variables significant during bivariate analysis at a p-value less than 0.05 were introduced in the regression model to appreciate the adjusted effect and derive the adjusted odds ratio (aOR) of each on the primary outcome, "risky sex" expressed as a dichotomous variable. A 95% level of confidence was applied throughout.

### Ethical considerations

This study was approved by the National AIDS and STI Program of Togo (Ref N° 098/2013/MS/DSSP/PNLS-IST). In Togo, the National AIDS and STI Program acts as an ethics committee. We obtained written consent from study participants. For each person interviewed, the objectives, benefits to participate in the survey and progress of the investigation were clearly stated as well as their right to interrupt the interview without justification. Participants were asked to sign an informed consent form after a verbal explanation of this information by the investigating officer in the preferred language of the participant. Participant identification information was not collected in order to maintain anonymity and confidentiality of study participants and the information they disclosed.

## Results

In total, 291 PLWHA on ART, including 201 (69.1%) women, were surveyed. The mean age of respondents was 37.27 ± 9.32 years (range: 18–68 years). The majority of patients were more than 35 years old (53.6%), were in a couple relationships (67.0%) and had at least primary level of education (72.2%); while 33.3% of PLWHA lived in rural areas (Table 1).

**Table 1:** Demographics, clinical, behavior, and treatment outcomes of PLWHA on ART

**Characteristics**	**Number of PLWHA (N)**	**Percentage (%)**
**Age**		
*Under 25 years*	26	8.9
*25-35 years*	109	37.5
*Over 35 years*	156	53.6
**Sex**		
*Male*	90	30.9
*Female*	201	69.1
**In couple**		
*No*	96	33.0
*Yes*	195	67.0
**Educational level**		
*No education*	81	27.8
*Primary school*	105	36.1
*Secondary school*	84	28.9
*High school*	21	7.2
**Place of residence**		
*Urban*	194	66.7
*Rural*	97	33.3
**ART duration**		
Less than *1 year*	33	11.3
*1 to 3 years*	126	43.3
*More than 3 years*	132	45.4
**Sexually active**		
*Yes*	217	74.6
*No*	74	25.4
**Risky sex**		
*No*	142	65.4
*Yes*	75	34.6
**Alcohol consumption before sex**		
*Yes*	32	14.7
*No*	185	85.3
**Disclosure HIV-status to regular sexual partner**		
*Yes*	131	60.4
*No*	86	39.6
**Current WHO’s stage**		
*Stage I*	23	7.9
*Stage II*	132	45.4
*Stage III*	106	36.4
*Stage IV*	30	10.3
**CD4’s count (last check-up)**		
*CD4 ≤ 350*	116	39.9
*CD4 > 350*	175	60.1
**Adherence to ART**		
*No*	73	25.1
*Yes*	218	74.9

With regards to sexual behavioral, 217 (74.6%) PLWHA surveyed were sexually active since initiating ART. Among them, women were less sexually active compare to men (66.7% versus 92.2%, p <0.001). Of the 217 sexually active patients, 34.6% reported having unsafe sex, 14.7% drank alcohol before sex and 60.4% had disclosed their HIV status to their regular sexual partners (Table 1).

On clinical and therapeutic level, 45.4% of PLWHA were receiving ART for more than three years, 46.7% were in stage III or IV of the WHO’s classification at the time of the survey, 60.1% had a CD4’s count ≥ 350 cells/mm3 and 74.9% had good adherence to ART (Table 1).

In bivariate analysis, PLWHA having unsafe sex was associated with the level of education of the participant (p = 0.016), duration of treatment (p = 0.001), alcohol consumption before sex (p = 0.005), disclosure of HIV status to their regular sexual partners (p = 0.002) and non-adherence to ART (p = 0.000) (Table 2).

**Table 2:** Risky sex according to patient’s characteristics

**Characteristics** ***, n***	***Having risky sex***		
	***No (%)***	***Yes (%)***	***P-value***
**Age**			0.132
*Under 25 years (14)*	71.4	28.6	
*25-35 years (86)*	73.3	26.7	
*Over 35 years (117)*	60.0	40.0	
**Sex**			0.347
*Male (83)*	69.9	30.1	
*Female (134)*	63.6	36.4	
**In couple**			0.377
*No (57)*	70.9	29.1	
*Yes (160)*	64.4	35.6	
**Educational level**			0.016*
*No education (53)*	52.8	47.2	
*Primary school (80)*	63.8	36.2	
*Secondary school (65)*	73.0	27.0	
*High* school *(19)*	89.5	10.5	
**Place of residence**			0.290
*Urban (148)*	63.7	36.3	
*Rural (69)*	71.0	29.0	
**ART duration**			0.001*
Less than *1 year (18)*	94.4	05.6	
*1 to 3 years (89)*	53.9	46.1	
*More than 3 year (108)*	71.3	28.7	
**CD4’s count (last check-up)**			0.454
*CD4 ≤ 350 (93)*	68.8	31.2	
*CD4* > *350 (124)*	63.9	36.1	
**Current WHO’s stage**			0.084
*Stage I (21)*	85.7	14.3	
*Stage II (93)*	67.7	32.3	
*Stage III (86)*	62.8	37.2	
*Stage IV (15)*	46.7	53.3	
**Alcohol consumption before sex**			0.005*
*Yes (32)*	43.3	56.7	
*No (185)*	69.7	30.3	
**Disclosure HIV-status to regular sexual partner**			0.002*
*Yes (131)*	74.0	26.0	
*No(86)*	53.6	46.4	
**Adherence to ART**			0.000*
*No (48)*	34.8	65.2	
*Yes (169)*	74.6	25.4	

*Statistical significant with p-value <0.05.

During multivariate analysis, four factors remained significantly associated with unsafe sex. PLWHA with poor adherence to ART were 2.6 times more likely to have unsafe sex (p = 0.014). In addition, PLWHA who consumed alcohol before sex were about 3.6 times more likely to have unsafe sex (p = 0.044). PLWHA with a higher level of education were less likely to have unsafe sex. PLWHA on ART for more than one year were more likely to have unsafe sex (Table 3).

**Table 3:** Effect of patient characteristics on risky sex

**patient characteristics**	**aOR**	**95% CI for aOR**	***p-value***
**Educational level**			
*No education*	Ref	*-*	***-***
*Primary school*	0.34	[0.15; 0.79]	0.011*
*Secondary school*	0.23	[0.09; 0.60]	0.003*
*High level school*	0.10	[0.02; 0.52]	0.006*
**ART duration**			
Less than *1 year*	Ref	-	*-*
*1 to 3 years*	27.08	[3.15; 233.09]	0.003*
*More than 3 years*	10.87	[1.29; 91.84]	0.028*
**Alcohol consumption before sex**			
*No*	Ref	-	**-**
*Yes*	3.56	[1.31; 9.64]	0.013*
**Disclosure HIV-status to regular sexual partner**			
*Yes*	Ref	-	**-**
*No*	1.97	[0.97; 3.99]	0.059
**Adherence to ART**			
*Yes*	Ref	-	**-**
*No*	2.59	[1.21; 5.54]	0.014*

Pseudo-R^2^ of the regression model = 20.24%.

*Statistical significant with p-value <0.05.

## Discussion

The study noted that four factors were associated with sexual risk behaviors among PLWHA on ART attending the regional hospital of Sodoké. These were: level of education, alcohol consumption before sex, duration of ART and non-adherence to ART.

In our study, 74.6% of the PLWHA on ART were sexually active. This proportion is higher than those reported by Bajunirwe et al. [8] in Uganda (51.4%), and Pearson et al. [15] in Mozambique (64%). The initiation of ART by improving the quality of life of PLWHA may have contributed to a resumption of sexual activity in these patients. Furthermore, of the 217 PLWHA who were sexually active, 34% had risky sex. This poses a risk of spread of HIV infection in the general population. Other studies in some developing countries have reported similar results [7,10,16-18]. This situation can be explained by reduced access to secondary prevention tools including condoms [7,16], but also the desire for procreation among PLWHA, especially in Africa where there are enormous social pressures to have children [19].

Our study shows that PLWHA who had poor adherence to ART were 2.6 times more likely to have unsafe sex. This is consistent with findings in a meta-analysis conducted in sub Saharan Africa where, Berhan et al. [11] attributed the positive changes in sexual behavior of PLWHA to their good adherence to ART. Promoting adherence to ART may, therefore, help prevent the spread of HIV infection in the population especially HIV infection with resistant viral strains.

The risk of having unsafe sex increased with the duration of ART. This suggests relaxation in the observance of preventive behaviors over time by PLWHA on ART. This observation was also reported by other authors in sub-Saharan Africa [11,20]. These studies mention that PLWHA, who begin ART usually, live with the symptoms of AIDS, decreasing sexual desire and increasing reluctance to engage in risky sexual practices. Contrary to these studies and ours’, other studies conducted in sub-Saharan Africa [16,18] have shown that the duration of ART was associated with safe sexual behaviors because of the effect of continuous counseling provided to these patients.

As in our study, other authors have noted that higher levels of education are protective against risky sexual behavior [10,21]. In fact, education promotes good life hygiene and a good perception of the risk of secondary transmission of HIV.

Finally, the consumption of alcohol before sex, although relatively low in our study (14.7% of sexually active PLWHA) was a good predictor of risky sexual behavior. The association between alcohol consumption and unsafe sex has been reported by other studies in sub-Saharan Africa among PLWHA on ART [8,22], and in other population groups [23]. Indeed, alcohol inhibits individuals’ perception of risk of HIV transmission and prevention behaviors.

### Limitations

This study was subject to a number of limitations. Firstly the sample may not be representative of the whole country. Sexual behavior likely differs substantially across Togo, which has a diversity of cultures and religions. Secondly, we relied on self-reported sexual behavior collected through an interview, which may underestimate proportion of risky sex. Finally, the definition of concepts such as unsafe sex and adherence to ART can vary from one study to another.

## Conclusion

There was a high prevalence of unsafe sex among PLWHA receiving ART at the regional hospital of Sokodé. We identified four factors associated with sexual risk behaviors which were: low education level, non-adherence to ART, alcohol consumption before sex and the duration of ART. These results should be confirmed by a national study with a longer follow-up of PLWHA on ART. However, the results of our study may provide clues to the national program against HIV/AIDS to conduct relevant and targeted interventions for secondary prevention of sexual transmission of HIV among PLWHA on ART in the central health region. The national program against HIV/AIDS should enhance health promotion to mitigate unsafe sexual behavior in this population group. Greater sensitization, both at population level and at health facilities, on the advantages of safe sex by using condoms consistently need to be reinforced.

## Abbreviations

aOR: Adjusted odds ratio; ART: Antiretroviral therapy; PLWHA: People Living With HIV and AIDS.

## Competing interests

The authors declare that they have no competing interests.

## Authors’ contributions

IY was responsible for the conception of the study, participated to the study design, undertook the field study, conducted the data collection, analysis and interpretation, and wrote the manuscript. PMP was involved in the study design, supervised data collection and participated in data analysis. BS and DEL were involved in the data collection, analysis and interpretation. They have revised and finalized the manuscript. MSM, SS, DI and BL participated in data collection, data analysis and interpretation of results, and provided comments on the manuscript. PP was responsible for the overall scientific management of the study, for analysis and interpretation, and the preparation of the final manuscript. All the authors have read and approved the final manuscript to be submitted for publication.

## Pre-publication history

The pre-publication history for this paper can be accessed here:

http://www.biomedcentral.com/1471-2458/14/636/prepub
